# Describing Point of Entry into Care and Being Lost to Program in a Cohort of HIV Positive Pregnant Women in a Large Urban Centre in Uganda

**DOI:** 10.1155/2017/3527563

**Published:** 2017-04-02

**Authors:** Rachel Musomba, Frank Mubiru, Shadia Nakalema, Hope Mackline, Ivan Kalule, Agnes N. Kiragga, Rosalind Parkes Ratanshi, Barbara Castelnuovo

**Affiliations:** ^1^Infectious Diseases Institute, Makerere University, Mulago Hospital, Kampala, Uganda; ^2^Cambridge Institute of Public Health, Cambridge, UK

## Abstract

*Introduction*. We aim to describe the time of entry into care and factors associated with being lost to program (LTP) in pregnant women on Option B Plus in an integrated HIV and antenatal care (ANC) clinic in Uganda.* Methods*. We included all pregnant women enrolled into the integrated HIV-ANC clinic from January 2012 to 31st July 2014, while the follow up period extended up to October 30th 2015. LTP was defined as being out of care for ≥3 months.* Results*. Overall 856 women were included. Only 36.4% (86/236) of the women were enrolled in the first trimester. Overall 69 (8.1%) were LTP. In the multivariate analysis older women (HR: 0.80 per five-year increase, CI: 0.64–1.0, and *P* = 0.060) and women on ART at the time of pregnancy (0.58, CI: 0.34–0.98, and *P* = 0.040) were more likely not to be LTP. Among women already on ART at the time of pregnancy no factor was associated with LTP.* Conclusion*. Our results suggest the need for interventions to enhance prompt linkage of HIV positive women to HIV services for ART initiation and for increased retention particularly in young and ART naive women.

## 1. Background

In many developed countries, paediatric HIV has been virtually eliminated [1]. The United Nations programme on HIV/AIDS (UNAIDS) estimates that currently 330,000 children are infected with HIV/AIDS worldwide, 90% of whom are living in Sub-Saharan Africa [2, 3]. Mother-to-child transmission (MTCT) is the primary mode of HIV infection in children either during pregnancy, labor, and delivery or through breast feeding [4–6]. Several studies have proven that antiretroviral drugs decrease HIV transmission from the mother to the baby [7–11]. Particularly, the use of triple antiretroviral (ART) therapy during pregnancy has been proven to be highly effective in reducing mother-to-child HIV transmission from 15% up to 45% with no treatment at all to below 5% [12].

To attain virtual elimination of paediatric HIV, programs in resource limited settings have scaled up integrated prevention of mother-to-child transmission (PMTCT) programs to identify and enroll HIV infected pregnant women into care and treatment services [13–16].

Since 2012, the WHO recommends immediate start of lifelong ART constituted by tenofovir, emtricitabine, and efavirenz, for all HIV positive pregnant and breast feeding women presenting into care regardless of their CD4 count or WHO clinical staging; this strategy is also known as Option B Plus [17].

Despite programs' efforts to scale up Option B Plus strategy by 50% coverage [18] and with up to 68.1% women starting ART during antenatal care and after delivery in Sub-Saharan Africa, the effectiveness of this prevention strategy to move towards an HIV free generation [19] is undermined by high rates of being lost to program (LTP) [20]. The rates of LTP are particularly alarming in the postpartum period (up to 50%) [21, 22].

So far most of the national programs in Sub-Saharan Africa, while challenged with low retention in care of mothers and their infants, are limited with interventions to improve retention into care and reduce LTP [23].

In Uganda, routine HIV screening is recommended for all women presenting for antenatal or delivery care in all public health facilities [24]; women who test HIV positive are offered, in addition to antenatal care (ANC), Option B Plus during prenatal and postnatal visits up to 18 months after delivering and are thereafter referred to HIV care services. However an earlier evaluation from public clinics in Kampala, the capital city, revealed alarming rates of being lost to follow-up between 25% and 58.8% [25].

At the Infectious Diseases Institute (IDI), Kampala, Uganda, an HIV centre of excellence, in order to increase retention into care and to avoid referrals to and from ANC services, with potential loss from care, an integrated HIV-ANC clinic was implemented in 2012. The main objective of this study was to describe the time of entry into care into the integrated HIV-ANC clinic and investigate factors associated with being lost to program.

## 2. Methods

### 2.1. Study Setting and Population

The Infectious Diseases Institute (IDI), Makerere University, is an HIV centre of excellence [26] located in Mulago Teaching Hospital in Kampala with over 8,000 HIV positive individuals receiving care. The IDI clinic began providing HIV care in 2002, while free antiretroviral treatment has been provided since April 2004. In 2012 an integrated HIV-ANC clinic was put into service, where all pregnant women receive antenatal and HIV care including Option B Plus.

### 2.2. The Integrated HIV-ANC Clinic

Women suspected to be pregnant on the basis of the date of their last menstrual period receive a targeted pregnancy confirmatory test, and if found positive they are referred to the integrated HIV-ANC clinic, while new HIV positive pregnant or breast feeding women referred to IDI are directed to the HIV-ANC clinic immediately at enrolment into care. In this clinic, pregnant women are offered iron and folic acid supplementation, antiretroviral drugs, prevention and management of opportunistic infections, education on obstetric practices, especially during labor and delivery, and counseling on infant feeding options. Mothers already on ART and those ART naive are prepared to start or switch to tenofovir, emtricitabine, and efavirenz as part of Option B Plus. At the time of this analysis ART was monitored using CD4 counts; viral load testing was not routinely available.

The clinic is staffed by a trained team of 3 medical officers, 2 nurses, and a nurse-counselor; it is supervised by a senior medical officer and assisted in logistic and health education tasks by a peer supporter.

### 2.3. Data Collection

At IDI patients' clinical information is captured into an in-house built provider-based electronic medical record EMR system called Integrated Clinic Enterprise Application (ICEA) [27]. The system generates automated queries to eliminate omission of mandatory fields and has internal consistency checks which ensure that the data entered is accurate. Within the main patient's management application, information on pregnant women and their babies can be entered in a dedicated module.

The Option B Plus ICEA module captures data before and after delivery which include information on whether the pregnancy was intended, gravity, parity, number of abortions, last normal menstrual period, expected date of delivery, and birth outcomes.

### 2.4. Statistical Analysis

In this analysis, we included all pregnant women who started ART in the HIV-ANC integrated clinic from 1st January 2012, the year Option B Plus was implemented, to 31st July 2014, while the follow-up period extended up to 30th October 2015. We used proportion to describe the point of entry into care (1st, 2nd, and 3rd trimester, on the day of delivery and postpartum) and the magnitude and reason of discontinuation from care (LTP, death, or transferred to another program). We used Kaplan-Meier survival analysis to estimate time to LTP. A woman was defined as* LTP* if she was alive but had not come back for her appointment for at least 3 months; women who had been transferred out of care but were attending another facility and receiving ART were not considered LTP.

Women were followed up from time of enrolment into the ANC to date of last clinic encounter or date of database closure for those still in care. We used Cox proportional hazards model methods to identify factors associated with LTP among women enrolled into the integrated HIV-ANC. Variables included in the model were age, WHO stage, parity, CD4 count, and ART status (ART naive versus on ART). Variables with a *P* value ≤0.25 in the unadjusted Cox proportional hazards model and those of clinical significance were included in the multivariate model. We also conducted a subanalysis including women already on ART at the time of pregnancy.

Analysis was performed using STATA 12.2, Texas, USA.

### 2.5. Ethical Statement

This study was approved and annually renewed by the School of Medicine Research and Ethics Committee, Makerere University Medical School (Reference number 2009-120), and the Uganda National Council for Science and Technology. The investigators obtained verbal or written consent waiver; all the information is analysed after stripping it of unique personal identifiers.

## 3. Results

Overall 856 pregnant women were included in the analysis; at the time of the enrolment into the HIV-ANC integrated clinic, the median age was 31 years (IQR: 26–35), and 302 (35.3%) were in WHO stage 3/4; the median CD4 count was 433 cells/*μ*L (IQR: 301–638), 236 (27.6%) were ART naive, and 329 (39.5%) had already had at least 2 pregnancies (Table 1).

**Figure 1:**
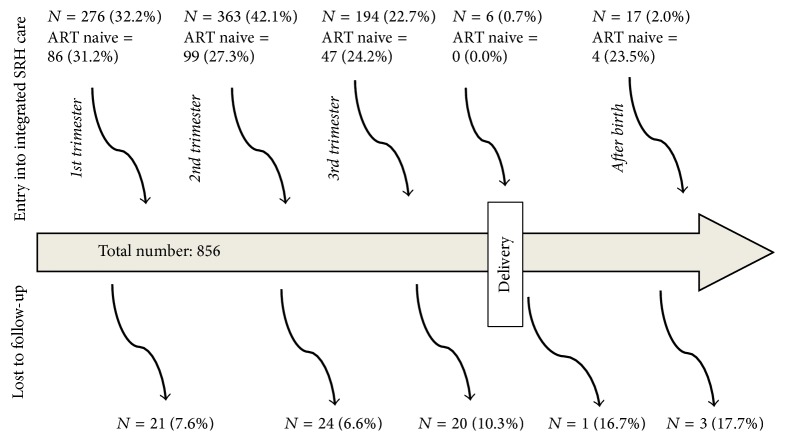
Showing point of entry and being lost to program by gestational age.

Figure 1 shows the time of entry into care in the integrated HIV-ANC clinic by gestational age and numbers of patients LTP. Only 32.2% (86/276) of the women were enrolled in the first trimester, and of these 86 (31.2%) were ART naive. Three hundred and sixty-three women (42.1%) were enrolled in the 2nd trimester, and of these 99 (27.3%) were ART naive; only 17 (2%) of patients were enrolled after birth, and among these 4 (23.5%) were ART naive.

The overall proportion of women LTP was 8.1% (69/856) with higher proportion of LTP observed in the postdelivery period (Figure 1).

There was a total follow-up time of 22262 person months. The median time to loss to follow-up was 12.4 (4.2–21.3) months and active patients had a follow-up time of 28.8 (20.7–37.5) months.

**Figure 2:**
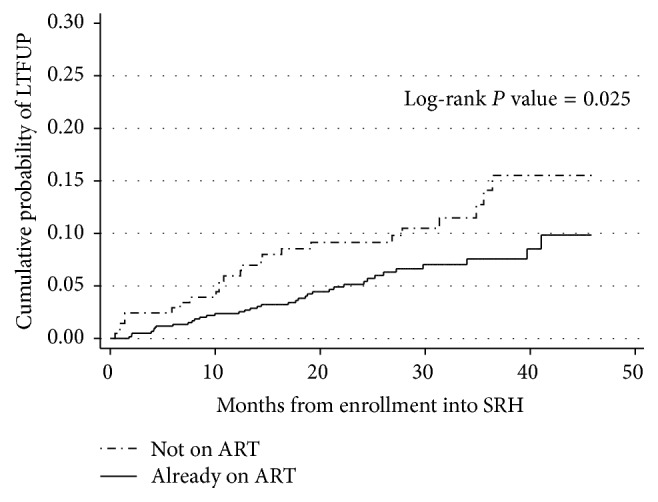
Showing cumulative probability of being lost to program by ART status.

Figure 2 shows the cumulative probability of being LTFU by ART status; women who were ART naive at the time of pregnancy had a higher cumulative probability (0.155, 95% CI: 0.102–0.232) of being LTFU as compared to those already on ART (0.085, 95% CI: 0.0587–0.123) (*P* = 0.025).

In the univariate analysis older age (HR: 0.77, CI: 0.62–0.96, and *P* = 0.019) being on ART at the time of pregnancy (HR: 0.56, CI: 0.33–0.94, and *P* = 0.0270) was associated with lower risk of being lost to program (Table 2).

In the multivariate analysis women of older age (HR: 0.80 per five-year increase, CI: 0.64–1.0, and *P* = 0.060) and women on ART at the time of pregnancy (0.58, CI: 0.34–0.98, and *P* = 0.040) remained factors associated with reduced LTFU. In the subanalysis that included only women who were not ART naive at the time of pregnancy, we found no factors associated significantly with LTP.

## 4. Discussion

In this study we described the point of entry and being lost to program in an integrated HIV-ANC clinic in Kampala, Uganda. We found that in our program rates of LTP are lower, as compared to other programs in in Sub-Saharan Africa; findings from Ethiopia demonstrated 16.5% [28] and Malawi 17% [29] of women LTP. Our results suggest that training dedicated staff and integration of services may lead to good levels of retention into care.

However in our program, similarly to what is reported from other countries [30, 31] despite overall good retention, only one third of ART naive women joined in the first trimester, with potential risk of transmission to their infants.

In our study we found higher rates of LTP in younger women and in women who were ART naive at the time of pregnancy and in the postpartum period. The high rates of LTP among young women are likely to reflect the low retention into the cascade from testing HIV positive to being uninterruptedly on treatment observed in this group [32]. Several factors may contribute to this; for example, young people are less concerned about their health compared to older people, they tend to have more risky lifestyle due to a youthful sense of invulnerability, and they are usually healthier and often engage into activities that interfere with routine clinic scheduled visits [33–35]. Additionally, stigma related to pregnancy among young people can influence retention in care [36, 37].

We also found that women on ART at the time of pregnancy were less likely to be LTP. Our findings are consistent with those of previous studies which indicated that ART experienced patients are more likely to be retained in care [28].

We observed that LTP was higher during postpartum period. Similar findings were reported in a cohort study done in South Africa in which 49% of women had disengaged from care postpartum [21]. These findings are also consistent with results from other studies [21, 38, 39]. We can hypothesize that, with the decentralization of HIV services in including provision of antiretroviral drugs, a proportion of women self-transfer to facilities nearer to their home after delivery [40]. This is possible in order to reduce transport costs.

Lack of disclosure of HIV positive status has been associated with increased LTFU [23, 41] in women enrolled in PMTCT programs. We hypothesize that women who have not disclosed report to their partners that they visit monthly a health care facility for ANC, but not for HIV care; after delivering they may stop coming to the clinic since they cannot justify to their partners' monthly visits to a health care facility.

One limitation of this study was that the information was obtained from routinely collected data, which may pose challenges in terms of data quality. However a previous evaluation of our database showed high completeness and consistency of data [27]. Another limitation is that we did not have preintegrated HIV-ANC clinic data for comparison purposes, and therefore we cannot attribute the high retention demonstrated in this study entirely to HIV-ANC integrated clinic, as other measures have been implemented at the entire clinic level to improve retention in for all patients.

Although reports from observation studies in Sub-Saharan Africa seem to suggest that integration of ANC including Option B+ and HIV services improves retention, this needs to be validated in more rigorous studies [42].

## 5. Conclusion

While in our program we achieved good retention of pregnant women enrolled in the Option B+ program, integration of services may not be sufficient to reduce being lost to program among young women and those who are naive. Where available, for groups at risk of being lost to program, resources should be utilized for other interventions which have demonstrated to be effective in increasing retention.

## Figures and Tables

**Table 1:** Characteristic of pregnant women at enrollment in the integrated HIV-antenatal clinic.

Characteristics	*N* = 856
Age (years), median (IQR)	31 (26–35)
Stage of pregnancy, *n* (%)	
First trimester	276 (32.2)
Second trimester	363 (42.4)
Third trimester	194 (22.7)
On delivery	6 (0.7)
After birth	17 (2.0)
Already on ART, *n* (%)	620 (72.4)
WHO clinical stage 3/4, *n* (%)	302 (35.3)
Parity >2, *N* (%)	329 (39.5)
CD4 cells/*μ*L, median (IQR)	433 (301–638)
*n* (%) ≤350	288 (33.7)
351–500	219 (25.6)
>500	347 (40.6)

ART: antiretroviral treatment.

**Table 2:** Factors associated with loss to program using Cox Proportional Hazards model.

Characteristics	Unadjusted HR (95% CI)	*P* value	Adjusted HR (95% CI)	*P* value
Age per 5 year increase	0.77 (0.62–0.96)	0.019	0.80 (0.64–1.00)	0.060
Gestational age				
First trimester	1.00			
Second trimester	0.75 (0.40−1.41)	0.370	0.78 (0.42–1.46)	0.439
Third trimester	1.44 (0.77–2.69)	0.260	1.52 (0.80–2.82)	0.210
On delivery	3.45 (0.46–25.81)	0.230	4.22 (0.56–31.9)	0.163
After birth	2.00 (0.47–8.58)	0.350	2.20 (0.51–9.42)	0.290
ART status at enrollment				
ART naive	1.00			
On ART	0.56 (0.33–0.94)	0.027	0.58 (0.34–0.98)	0.040
WHO clinical stage				
I-II	1.00			
III-IV	0.91 (0.54–1.54)	0.730		
Parity				
≤2	1.00			
>2	0.82 (0.48–1.39)	0.454		
CD4 cells/*μ*L				
≤350	1.00			
350–500	0.98 (0.51–1.92)	0.980		
>500	0.97 (0.54–1.73)	0.910		
